# Ovulation and Menstruation, and Dr. Battey’s Operation of Normal Ovariotomy

**Published:** 1876-03

**Authors:** Ch. Rauschenberg

**Affiliations:** Atlanta, Ga.


### OVULATION AND MENSTRUATION,

### And Dr. R. Battey's Operation of Normal Ovariotomy.

By CH. RAUSCHENBERG, M.D., Atlanta, Ga.

When Dr. R. Battey, then of Rome, Ga., performed his operation of normal ovariotomy the first time, in August, 1872, his case was reported and discussed at this Academy of Medicine, and several of its members will recollect that my views of ovulation and menstruation, and the effect of Dr. Battey's operation upon these functions, were materially different from those of a large number of the members of the Academy, as well as Dr. Battey's. Discussions on this subject, as they afterwards occurred, appear in the Atlanta Med. and Surgical Journal, amongst the proceedings of the Atlanta Academy of Medicine. They show that I assail the correctness of those physiological principles which caused Dr. Battey to adopt the removal of the normal ovaries as a surgical remedy—to use his own language, "*with a view to establish at once the change of life, for the effectual remedy of otherwise incurable maladies.*" Several years further consideration and study of the physiology of the functions and the origin and nature of sexual life in the female, leave me still on the same physiological platform from which I have opposed this operation of normal ovariotomy as a means of establishing the so-called change of life at once, and of thus curing those obstinate diseases of the ovaries and womb, which we frequently see disappear or improve in females after the normal cessation of the functions of these organs, and I am firmly convinced that this operation is only indicated and admissible in a very few rare cases, but that the broad indication upon which its author bases its performance, as quoted above in his own language, is physiologically and pathologically untenable and wrong, and therefore inadmissible.

In accordance with a promise made to this Academy some time ago, and in the interest of medical science, but without the slightest desire to offend Dr. Battey personally, or to detract from his professional merits, I will endeavor to define the points of difference between Dr. Battey and his many supporters and myself; taking his articles on Normal Ovariotomy, contained in the ATLANTA MEDICAL AND SURGICAL JOURNAL, September, 1872 and April, 1873, as the exponents of his views.*

Dr. Battey labors in these articles to establish the following points: 1st. That the so-called change of life, consisting both of the cessation of the menses and the cessation of the menstrual impulse, will, according to the experience of the best authorities, remove many of the most obstinate troubles, particularly ovarian congestion, subacute inflammations, and the most chronic diseases of the womb. 2d. That menstruation depends upon ovulation alone, and that it therefore ceases to exist with the maturation and discharge of ova from the ovaries. 3d. That the surgical removal of the ovaries therefore brings on the change of life, the cessation of ovulation and menstruation, and therefore removes those diseases of the ovaries and womb, which are usually seen to disappear when that change takes place in the life of females.

Are these suppositions physiologically correct? Does the main physiological feature of the so-called change of life consist in the cessation of menstruation? Does a female every time when she menstruates also ovulate, or does menstruation occur also without ovulation, and have we any physiological right to look upon these occurrences as one and the same physiological process, in which ovulation represents the cause, menstruation the effect, and which becomes a physiological impossibility as soon as the ovaries are removed?

I am well aware that it is the received opinion of a majority of the medical world that menstruation is an impossibility without ovaries, because it is said never to occur in females with congenital malformation or absence of these organs. It has not been my good fortune to have had an opportunity of examining the records of medical literature so as to know how far the anatomi-

*This essay was read before the Atlanta Academy of Medicine at its second meeting in September, 1875.

cal deficiencies in such cases were confined to the ovaries alone, and how far the functional abnormities corresponded with them. I have seen no detailed description of the history and the post mortem appearances of the womb and ovaries in such cases, but only general statements. The question therefore arises in my mind: Is there a single case on record of a female who never menstruated, where it was established beyond a doubt that she had no ovaries at all, but a perfectly developed and histologically normal womb and sexual organs generally? I do not know this to be the case, and therefore confess my ignorance, trusting that members of the profession, who have access to a larger store of medical literature than myself, will dispel my doubts on this point. Let us, however, presume that such cases are on record, and that this opinion has been proven beyond a doubt by anatomical observation and clinical experience, does that prove that if we deprive the normally developed female, after the age of puberty, of her ovaries, that we disable her normally developed womb to perform its functions? Is that womb merely instituted because there exist ovaries? Is it a mere dependency, a mere vassal of these organs, working, so to say, merely and only at their bidding, or has it an independent functional capacity, and a destiny in relation to the entire organism and economy inherent within the cells of its tissues, whenever it arrives at maturity of development? Does its functional action only depend upon the existence of the ovaries, or does it occupy that mutual relationship to its kindred organs and to the entire female organism, and *vice versa*, they towards it, which constitutes the generally prevailing law of the animal organization in relation to the different organs it is composed of?

This law of physiology applies to the womb and the ovaries alike; they influence each other functionally, and again the central organs of the nervous system—the brain and spinal marrow as these do them, in such a manner that any quantitative or qualitative chemical or histological morbid change in either will, according to its peculiar character, influence the development or physiological action of one or all of the others in such a way as the laws of life necessitate it? If, then, one or the other of the two organs is congenitally imperfect or entirely absent, if one link in the chain of organs, constituting one physiological sphere of the system, is thus deficient from the beginning of life, may we not properly look for a train of certain unavoidable consequences in the histological development and the functional action of the others?

Can we, therefore, if the ovaries are congenitally absent or rudimental, logically expect a perfect histological development of the womb, or that reflex action through the spinal cord, backward and forward, which is so essential to the development of sexual life and the organs which serve its purposes? Can we, therefore, not only readily understand, but must we not rationally conclude that the congenital want of the ovaries will more or less interfere with that development of nerve influence upon the nerve centres, and from these upon the other sexual organs, which is necessary for their histological development and functional maturity? Can we therefore wonder if with this congenital absence of the ovaries, the womb itself, as well as the entire sexual system and life, fall more or less short of histological and functional maturity, and if menstruation does only occur imperfectly or not at all in such cases?

How does the case stand, however, if all these organs, with the cerebro-spinal centres, are already histologically developed, functionally matured, and sexual life, psychically and physically, established and in active operation, and the ovaries are then artificially removed? Can we then look for an entire and complete cessation of the functions of the womb in consequence of this removal?

If the womb, as I have already stated before, has, like every other organ of the human system, inherent within itself its own specific life and functional capacity, in conformity with and depending upon its specific histological structure and the specific life and vital qualities and action of the cells of its tissues, its functional life must continue as long as it remains itself anatomically intact, and the absence of the ovaries can only have a very gradual influence in modifying its functional activity, but can never, according to my conceptions of physiology, arrest them at once and entirely. It seems, according to the authorities of Dr. Battey, Meigs, Tyler Smith and others, that menstruation depends *alone* upon ovulation, or in other words, upon the impulse which the womb receives at that time from the ovaries.

Dr. Battey concludes that when this so-called ovarian impulse is gone, with the loss of the ovaries, the womb itself becomes a physiological nullity; it loses its entire functional capacity, and occupies no longer any relation to the organism, in the same manner as if it had been removed with the ovaries.

Now, gentlemen, what do we know about this ovarian impulse? Can anyone establish it as a fact, that it is primary in its nature, originating primarily and exclusively in the ovaries, or is there not the very strongest probability, amounting almost to a certainty, that it depends upon *a more general physiological force*, by whose presiding power over the organs of regeneration the harmonious co-operation of all their functional capacities is established for the accomplishment of the final objects for which they exist? I need not tell you that I am decidedly of the opinion that the ovaries and uterus are co-operative organs, each one, however, with a separate and distinct function; each one serving a distinct and different purpose, and that the functional action of each one, while it may be modified by nerve influence, by consensus from the other, is in no wise primarily and alone depending upon that of the other, but that both are servants of one great physiological law of the female organism, in order to accomplish the main physical destiny of the female, the reproduction of her species, the propagation of her race.

A further discussion of this subject will be unavoidably connected with the consideration of the physiology of ovulation, menstruation, and the so-called change, of life, and there I trust my views will be more clearly and fully defined; here I only desire to state finally, that the existence of an ovarian impulse, as the sole cause of menstruation, remains yet, to use very mild expressions, a hypothetical uncertainty, and, according to my views, a strong improbability.

I shall now endeavor to analyze the physiological theses upon which Dr. Battey bases, and by which he justifies, his operation. I do but seldom quote his own language, but any one examining his article on Normal Ovariotomy, from April, 1872, will admit that he first desires to establish, as I have already stated above *that the so-called change of life, which he says includes both cessation of the menses and cessation of the menstrual impulse, will remove many of the most obstinate troubles, particularly ovarian congestion, subacute inflammations, and the most chronic diseases of the womb.** No practitioner, of experience or fair information, will deny that certain diseases of the womb and ovaries and their appendages, and even diseases of the pelvic cavity, improve considerably, or disappear entirely, after the change of life. Why is this so?

As long as the walls of the capillaries of these organs, and the

*Atlanta Med. and Surg. Journal, vol xi., April, 1873. pp. 4 and 5.

blood-vessels of the pelvic cavity of the female, are subjected at regular monthly intervals to that blood pressure and expansion necessarily connected with the monthly flow of blood to these organs, morbid chronic congestions, hyperæmia and distension of blood-vessels already existing there, to-wit: oophoritis, endometritis, metritis, etc., with their relative consequences, leucorrhœa, prolapsus, and even more distant morbid conditions, such as hemorhoids, varicose veins, etc., receive what I am inclined to call a physiological support every month, and the female must, therefore, during that period of life, remain particularly prone to suffer from these and kindred diseases; while when this physiological cause of irritation, this physiological support, ceases with the change of life, the conditions for a restitution of the morbidly altered tissues set in, and an improvement, and often an entire recovery, take place.

The cessation of this monthly congestion to the pelvic organs, with the change of life, is undoubtedly the cause of this improvement. Dr. Battey proposes, by the removal of the ovaries at any period of life previous to that time, to bring on the same physiological change in the entire organism which usually takes place at the age of forty-five years of female life, after the sexual organs and the entire economy of the female organism have been serving the objects of reproduction for a period of thirty or thirty-five years. He says: "If the ovular theory of menstruation is to be accepted as an ultimate fact, the answer to my second question, to-wit: 'Was there rational ground for the belief that removal of the ovaries would bring on the change of life?' becomes as easy and as logical as the first. If the menstrual nisus be simply the result of ovulation, then the cessation of ovulation, the cause, necessitates the cessation of the effect of that cause, whether this cessation occur by reason of atrophy of the ovaries, by age, or their removal by the knife. It seems to me, therefore, that the question to be considered is: Are there reasonable grounds for the acceptation of this ovular theory?*

At the commencement of this article I have endeavored to express these conclusions of Dr. Battey in the second and third theses, which he desires to establish as physiological truths, and which amount to this: *Menstruation depends upon ovulation alone; it ceases to take place with the maturation and discharge of ova. The surgical removal of the ovaries checks ovulation, therefore*

*Normal Ovariotomy.—Atlanta Medical and Surgical Journal, Vol. XI. p. 5.

*menstruation; therefore brings on the change of life; therefore cures the diseases spoken of; therefore is a sovereign and universal remedy for these often otherwise incurable diseases.*

With all due respect to Dr. Battey, I must say that I consider this mode of reasoning and the conclusions arrived at decidedly empirical and erroneous.

I have shown above that in the diseases in which Dr. Battey expects an improvement from the change of life, and a cure from his operation, the cessation of the monthly congestion to the ovaries and womb and their appendages, causes that improvement.

Let us now inquire, Why does this congestion take place in the female organism from the age of puberty to the cessation of sexual life, and why does is cease at that time? Why is it that so much blood finds its way monthly during that period to the sexual organs of the female, and is from there discharged and wasted unless conception takes place? Why is it that a female, no matter how delicate she may be, can lose, for a period of thirty or thirty-five years, every month, a comparatively large quantity of blood without any detriment to her health—nay, why is it that her very health, her bodily and mental welfare, and almost her very existence, depend upon this monthly loss of blood as long as a state of pregnancy or subsequent lactation does not exist?

I need not tell you that the entire organization of the female during the period of sexual life is principally engaged in the accomplishment of the main object of female existence—*that of the reproduction of her species*—by the formation and maturation of the ova in the ovaries, and the nutrition and development of the fœtus in the womb. For the accomplishment of this object provisions are made for the preparation of a larger quantity of blood than she needs for her own nutrition, and also provisions by which this surplus amount of blood is trained off as long as the conditions under which it is needed within the female organization are not in existence. Its preparation, as well as its discharge, unless used for the purposes of gestation or lactation, are necessities of equal importance for the well-being and integrity of the female. Without the first no ovum can be detached, no fœtus matured; without the last no unimpregnated female can enjoy health. Ovaries without this production of surplus blood would serve no purpose, because the material for the nutrition and growth of the impregnated ovum would be wanting, and with it but without conception, or without its regular monthly discharge, female life would be an impossibility. Those who are not willing to admit this as a fact I simply remind of the alarming symptoms of congestion of important organs, which frequently follow sudden suppression of the menses, and also the frequent appearance of epistaxis, vomitus cruentus, hæmoptysis, phthisis, dropsy, epilepsy and hysteria in all its forms, and even insanity in females, with amenorrhœa and dysmenorrhœa, as well as of the fact that many cases of these diseases are depending upon constitutional quantitative or qualitative deficiencies, morbid conditions in the life *of the blood* of the individual, but not primarily in local diseases of the ovaries, as many of the gynæcological surgeons of our day are too much inclined to suppose.

Menstruation, therefore, was properly called by the ancient physicians, *signum et præsidium sanitatis*, the evidence and preserver of female health. Upon ovulation depends conception; upon the surplus blood preparation depends primarily the monthly congestion, the external, visible evidence of which we call menstruation, and upon the co-operation of the functions of the ovaries and womb the development of the Graafian vesicle to an ovum, and of that into a fœtus in the womb, if it should happen to become impregnated. Without ovulation, the female organism, in its normal state, cannot make use of this surplus blood without a disturbance of the general health, and therefore it is evident that in a female without ovaries the necessity for its discharge from the system increases instead of diminishing, unless the fact can be established that with the removal of the ovaries this production of surplus blood ceases also.

Is there a single physiological reason why this should be the case? Can any one, who has any conception of physiology at all, suppose for one moment that the entire vegetative life of the female, the processes of nutrition, chylification, sanguification, etc., with all their connections and influences, could primarily depend upon the functional activity of the ovaries, the development of Graafian vesicles and this periodical maturation of ova, could control with almost omnipotent power the entire female organization?

Readily do I admit that the irritability of the nerves of these organs is subject to a gradual rise and fall, which corresponds respectively with the periods of their greatest and smallest functional activity, each state occurring every twenty-eight days, and that the high degree of irritability and the resulting functional excitement of reflex action at the time of maximum functional activity becomes secondary, and in a degree instrumental, in increasing the monthly congestion of the sexual organs; but I can never admit that this monthly irritation of the ovaries constitutes the primary cause of the entire train of physiological conditions in the female system, which are necessary to establish the monthly flow of blood, much less that the first requisite, the production of blood in the female body, over and above what is needed for its own nutrition or its monthly discharge, will be arrested by their removal. I look upon sexual life and its processes and manifestations as the result of the perfect chemical and histological development of the solid and fluid constituents of the blood, nervous system and the sexual organs, and the resulting harmonious co-operation of their respective functions. The normal female attains this perfection at the age of ten to fifteen years. It comprises all the conditions for the accomplishment of all the objects of sexual life; but with the constant performance of functional duties, with the increasing waste of material and tissue, it commences to decline, and after a period of thirty or thirty-five years of constant activity these conditions cease to exist, and with this cessation the so-called change of life takes place.

Dr. Battey's operation of removing the ovaries, if it brings about, as he contends, the change of life, must bring on the same physiological status of the female organism which exists there after the natural, or rather the physiological, change of life. This conclusion is unavoidable. Hence Dr. Battey accomplishes these changes in the entire female organism with a few strokes of his knife, which, according to the plain laws of life, can only gradually take place, as the consequence of over a quarter of a century's functional activity. The blooming maiden of yesterday, lo! and behold, is a sedate matron to-day! What a wonderful process, by which the great law of nature, that of gradual development and gradual decline, is all at once set aside, and the female organism and its physiological manifestations forced to jump over a period of from ten, twenty, thirty, or even more years, and made to run its course smoothly, as if it had existed and lived that long in reality. What a pity that we cannot devise some surgical operation by which we could produce such a change, not forward, but backward, and rejuvenate people, and particularly females, by that many years. Perhaps it would gain popularity faster still than normal ovariotomy has.

These remarks are made, not with a desire to ridicule, but to present plainly the full absurdity of the conclusions which unavoidably follow the acceptance of the proposition that the surgical removal of the normal ovaries accomplishes the change of life.

The change of life is brought on by the gradual decline of those conditions in the female organization, by whose co-operation the functional integrity of the reproductive organs is established and maintained, and the maturation and discharge of ova in the ovaries, and the nutrition, growth, and full development of the impregnated ovum in the womb, accomplished. The decline of these conditions and their final cessation is as much a physiological necessity as their first development at the age of puberty.

Menstruation, the periodical discharge of the blood, has nothing to do with the accomplishment of the objects of sexual life. It is only the visible evidence of the fact that the blood necessary for the purposes of reproduction has been prepared, has found its way to the ovaries and womb, and has not all been needed there in the absence of the conditions requiring the development of an impregnated ovum. This discharge is a physiological necessity for the health of the female, and only the consequence of, but not the physiological process itself, that requires our main consideration. This process, to-wit: the periodical congestion and hyperæmia of the ovaries and womb, in order to take place requires, primarily, a quantity of blood in the female larger than is necessary for her own nutrition and sustenance, and secondarily, a healthy state of the nervous system and the sexual organs.

The preparation of the blood in the female necessary for this periodical congestion cannot depend upon ovarian action. It is simply the consequence of the specific typical development of the female organism at the age of puberty. It goes on during the entire period of sexual life, and results in the monthly discharge, as long as impregnation does not take place; in the development of the fœtus, after pregnancy. In a few instances of the latter, although the maturation and discharge of ova from the Graafian vesicles, and with it the so-called ovarian impulse, ceases during that state, menstruation still continues, and has been known to continue up to the seventh month of this condition. The writer himself knows one female in this city, and has delivered her several times, who invariably menstruates regularly for seven months during pregnancy.

These instances furnish at least formidable cause for doubting Dr. Battey's position, and reasonable evidence that the periodical congestion of the womb and ovaries is possible without the so-called ovarian impulse, and does take place whenever a quantity of blood greater than is required for the sustenance of the mother alone, or even of the mother and fœtus together, is prepared and contained within the system, or in other words, that menstruation occurs without ovulation. In other cases, where the amount of blood is barely sufficient to sustain reproductive life—for instance in very young, or anæmic, or chlorotic subjects, ovulation and pregnancy take place without menstruation ever making its appearance, which means simply that the congestion has occurred, but has, up to that time, only furnished the amount of blood necessary for the process of gestation.

If it is sound physiology to conclude that the preparation of this surplus blood is not depending upon the ovarian impulse is it reasonable to suppose that the nerve action of the ovaries, the periodical monthly excitement of these organs, is the primary cause of their periodical congestion, or is it logical and in accordance with sound physiology to look upon the monthly plethora of the system and the subsequent increase of the blood pressure in the capillaries generally, and the sexual organs in particular, and the irritation of the vaso-motor, and trophic nerves of these organs, necessarily resulting therefrom, as the primary cause of the periodical sexual excitement and functional activity of the female organism, which either terminates in the normal development of a fœtus, or the monthly discharge of blood, by which the plethora of the system, the blood pressure, the irritation of the ovaries and womb, the general orgasm is relieved, until after twenty-eight days the increase in the quantity of the blood reaches again its maximum, and gives rise to the same process of local and general excitement.

I am decidedly of the opinion that this explanation of the periodical excitement of sexual life is more rational than the idea that the ovaries, as the producers of the ova, possess within themselves an independent primary nerve power, which is within itself powerful enough to bring on this production of surplus blood and this monthly congestion, independent of any other outside co-operation of the female organism, and that without the action of this nerve power the monthly congestion will not occur.

I do not deny the development of a higher degree of nerve power within the ovaries during the maturation and discharge of ova, which will of course exercise its influence upon the neighboring organs and the female system generally; but I do not admit that the vegetative life of these organs, the gradual development of ova, the development of a deciduous membrane within the womb, and the congestion of blood to these organs, by which these processes become possible, is depending upon anything else, primarily, but the mechanical and chemical impetus imparted upon the capillary walls and the vaso-motor nerves of the tissues, resulting necessarily from the periodical reproduction within the female system of a larger quantity of normal blood than she needs for her own purposes of nutrition. I do also not deny the power of the ovaries to produce, to receive and to convey impressions through their nerves to neighboring organs and to the system generally, but I cannot see any necessity to accept the idea of the existence of a so-called menstrual impulse as the primary and only cause of the monthly congestion of the ovaries and womb, and its results. With the denial of the existence of this menstrual impulse, as the cause of the monthly congestion and menstrual flow, I deny, necessarily, also the possibility of effecting the change of life by the surgical removal of the ovaries and the cure of the diseases intended thereby.

Why do the advocates of this ovular theory of menstruation hold that a female can never menstruate without ovulating; that the two processes belong together, and are identical? Why do they call any discharge or flow from the sexual organs, no matter how regular it occurs, a pseudo-menstrual discharge, a metrostasis? True enough, they are both the result of one and the same physiological process within the female organization, but different functional manifestations of two different organs, for distinct purposes, serving one final object; but they certainly do not occupy the relationship of cause and effect to each other.

Bischoff's discovery of the periodical maturation and discharge of ova from the Graafian vesicles, connected, on account of the sameness of their periodicity, the two processes of ovulation and menstruation together as one and the same in the mind of physicians, and gave rise to the confusion yet existing amongst the profession in relation to this subject.

The amount of the menstrual flow with each monthly congestion depends upon the amount of blood the female is able to prepare for the purpose of reproduction. She may, therefore, as already stated above, ovulate and become pregnant without menstruating; she may menstruate sparingly, moderately, or copiously, and she may continue to menstruate a longer or shorter time after pregnancy. Numerous instances of each kind can be found on record.* These facts, as far as they demonstrate the occurrence of ovulation without menstruation, you are willing to receive; but most of you will still ask: Does menstruation occur without ovulation? I ask, Why should the ovaries not fail sometimes to act normally, in consequence of this congestion, as much so as the womb does sometimes? Graafian vesicles, if the ovaries are histologically imperfect, may fail to be developed, or may only undergo imperfect development, and never reach the surface and burst. All this may take place, but I cannot see a single physiological reason why, in case of such histological deficiencies, unless they are congenital, the flow of blood to the womb should at once be checked, or cease to produce there the same physiological results that it usually does. I admit that ovulation is included when we speak of that physiological process which finally terminates in menstruation; but I do not admit that this process does not occur without the development of ova, or that it depends primarily upon this development. If menstruation does not occur without ovulation, why do pathological anatomists not always find a fresh corpus luteum on the ovaries of females who died during menstruation? The fact that they frequently do not find it cannot be denied. Bischoff himself, Rœlliker, and others have repeatedly met with such instances.

Furthermore, if menstruation cannot occur without ovulation, why does it occur after pregnancy? The maturation and discharge of new ova cease as soon as pregnancy takes place. Prof. E. Schultze† says: "Not a single exception from this rule has ever been established by the post mortem condition of the ovaries of pregnant or lying-in women." All pathological anatomists agree that they always can easily discover the corpus luteum verum of the existing pregnancy, but not a single later mark of

*Hogg.—Notes on Menstruation, Med. Times and Gazette, Nov. 4, 1871.

†Zwillings schwangerschaften. Volkmans Klinische Vorträge, No. 34, p. 310.

a ruptured vesicle. If the greater development of a corpus luteum, the ovum of which becomes impregnated, is owing to the more permanent and larger blood-supply of the ovaries during pregnancy, there is no reason why any vesicles, which rupture afterwards should not undergo the same change as the one to which the pregnancy is owing, and should become corpora lutea vera. But never more than one of them, and not a mark of comon late corpora lutea, as they appear when their ova do not become impregnated, has ever been observed under such circumstances.

Slavianski, in his late researches,* arrived at the conclusion that Graafian vesicles are continually engaged in a process of development and maturation during childhood as regular as during the age of puberty; but do not reach the surface of the organ, and therefore do not burst, but undergo a process of retrogressive metamorphosis. If the monthly congestion is caused by the irritation of the ovaries by the ripening of ova, the so-called ovarian impulse, and its effect upon the womb and the system generally, why does this process, then, if Slavianski's observations are correct, not commence long before the age of puberty? This is not the case, because ovarian functional action alone is not sufficient, as I have been endeavoring to illustrate during the entire course of this essay, to establish sexual life; but it requires the full maturity of the blood, the nervous system and the sexual organs, and their harmonious co-operation to establish the combination of functional activity constituting perfect sexual capacity.

Thus the occurrence of menstruaton without ovulation is at least more than a probable occurrence, and the conclusion of Wells and others, adopted by Dr. Battey, that all these monthly discharges of blood, no matter how regular they occur, where the possibility of ovulation is or seems excluded, shall be called cases of mere pseudo-menstruation, or metro-stassis, does, in reality, not change the actual fact of their occurrence, and gives us not the slightest physiological reason why they should occur and should not be considered cases of menstruation in the full sense of the word.

You will now want to know why, if my theory of ovulation and menstruation is correct, the menstrual flow has ceased in some cases where the ovaries were removed in their normal state. I cannot give you an explanation of this occurrence upon the basis of the general principles I have advocated in this paper,

Archiv de Physiologie, 1874.

any more than the advocates of the ovular theory can explain why it did not cease in other such cases; but I am confident that a close investigation of their history and nature, where it can be had, will frequently furnish a reason. The fact that it did not cease for a long time, and has perhaps not ceased yet, in Dr. R. Battey's Rome case, I suppose remains also unexplained.

Can the results of the surgical removal of diseased ovaries prove anything for or against the hypothesis that menstruation depends upon ovulation?

If there is normal ovarian tissue still in existence in such diseased ovaries, which happens to remain in consequence of only partial removal by operation, menstruation, according to the ovular theory, must continue; if removed it must cease. According to my theory it will go on in either case, unless other circumstances cause its cessation. As no data are on hand in any of these cases to decide upon these points, they furnish no evidence for or against either theory.

These, and still more so the cases where the ovaries are entirely disorganized, where no normal ovarian tissue remains in existence, where, therefore, in accordance with the ovular theory, menstruation ought not to exist, even before the operation, and where a process of morbid development has been going on for a longer or shorter period of time, admit undoubtedly of the following question, to-wit: Would such a process of morbid nutrition within the ovaries, existing during a number of years, not establish a degree of local irritation, hyperæmia, consumption, and consequent waste of nutritive material, that the climacteric period, the time during which the female organism furnishes the increased blood supply for the purposes of reproductive action, would be considerably shortened? Might we then, in such cases, after the removal of the diseased ovaries, and of the morbid irritation, not expect, in some cases, an immediate, or at least an earlier cessation? This appears to me more than probable; but as the history of these cases has not been written with a view to these points, our means to decide upon the question before us, from the results of ovariotomy, are quite deficient, and I therefore cannot admit them as reliable evidence for or against my theory.

The consideration of the pathology of ovulation and menstruation upon the basis of the physiology of these processes, as set forth in this paper, I must necessarily defer to a future time, on account of the great length which it has already assumed.

